# Size- and Stereochemistry-Dependent Transcriptional Bypass of DNA Alkyl Phosphotriester Adducts in Mammalian Cells

**DOI:** 10.3390/dna2040016

**Published:** 2022-10-05

**Authors:** Ying Tan, Jiabin Wu, Garrit Clabaugh, Lin Li, Hua Du, Yinsheng Wang

**Affiliations:** 1 Environmental Toxicology Graduate Program, University of California Riverside, Riverside, CA 92521-0403, USA; 2 Department of Chemistry, University of California Riverside, Riverside, CA 92521-0403, USA

**Keywords:** DNA alkylation, transcriptional mutagenesis, DNA damage

## Abstract

Environmental, endogenous and therapeutic alkylating agents can react with internucleotide phosphate groups in DNA to yield alkyl phosphotriester (PTE) adducts. Alkyl-PTEs are induced at relatively high frequencies and are persistent in mammalian tissues; however, their biological consequences in mammalian cells have not been examined. Herein, we assessed how alkyl-PTEs with different alkyl group sizes and stereochemical configurations (*S*_P_ and *R*_P_ diastereomers of Me and *n*Pr) affect the efficiency and fidelity of transcription in mammalian cells. We found that, while the *R*_P_ diastereomer of Me- and *n*Pr-PTEs constituted moderate and strong blockages to transcription, respectively, the *S*_P_ diastereomer of the two lesions did not appreciably perturb transcription efficiency. In addition, none of the four alkyl-PTEs induced mutant transcripts. Furthermore, polymerase η assumed an important role in promoting transcription across the *S*_P_-Me-PTE, but not any of other three lesions. Loss of other translesion synthesis (TLS) polymerases tested, including Pol κ, Pol ι, Pol ξ and REV1, did not alter the transcription bypass efficiency or mutation frequency for any of the alkyl-PTE lesions. Together, our study provided important new knowledge about the impact of alkyl-PTE lesions on transcription and expanded the substrate pool of Pol η in transcriptional bypass.

## 1. Introduction

Exposure to endogenous and exogenous DNA damaging agents can compromise genomic integrity in human cells [1]. Unrepaired DNA lesions may impede DNA replication and transcription, and elicit mutations in these processes, which may contribute to the development of cancer and other human diseases [1]. Alkylation is a major form of DNA damage, and it can arise from metabolism, environmental exposure, and cancer chemotherapy [2–5].

Understanding the implications of these lesions in the etiology for developing human diseases requires knowledge about how alkylated DNA lesions inhibit DNA replication and transcription, how they induce mutations in these processes, and how they are repaired. Such knowledge may also facilitate the development of better strategies for cancer chemotherapy. In this vein, modulating the activities of translesion synthesis (TLS) and DNA repair is known to affect the anti-tumor activities of cancer chemotherapeutic agents [5–9]. Moreover, combination therapy, where DNA alkylating agents are used in conjunction with small-molecule inhibitors for DNA damage response or repair, is now in clinical trials for cancer treatment [5].

Depending on the nature of the alkylating agents involved, the size of the alkyl groups adducted to nucleobases can vary substantially. For example, some widely prescribed anticancer drugs can elicit methylation and chloroethylation of DNA [3,6] and metabolites of some tobacco-derived *N*-nitrosamines can lead to the adduction of pyridyloxobutyl (POB) and pyridylhydroxybutyl (PHB) groups with DNA [10].

**Figure 1.**
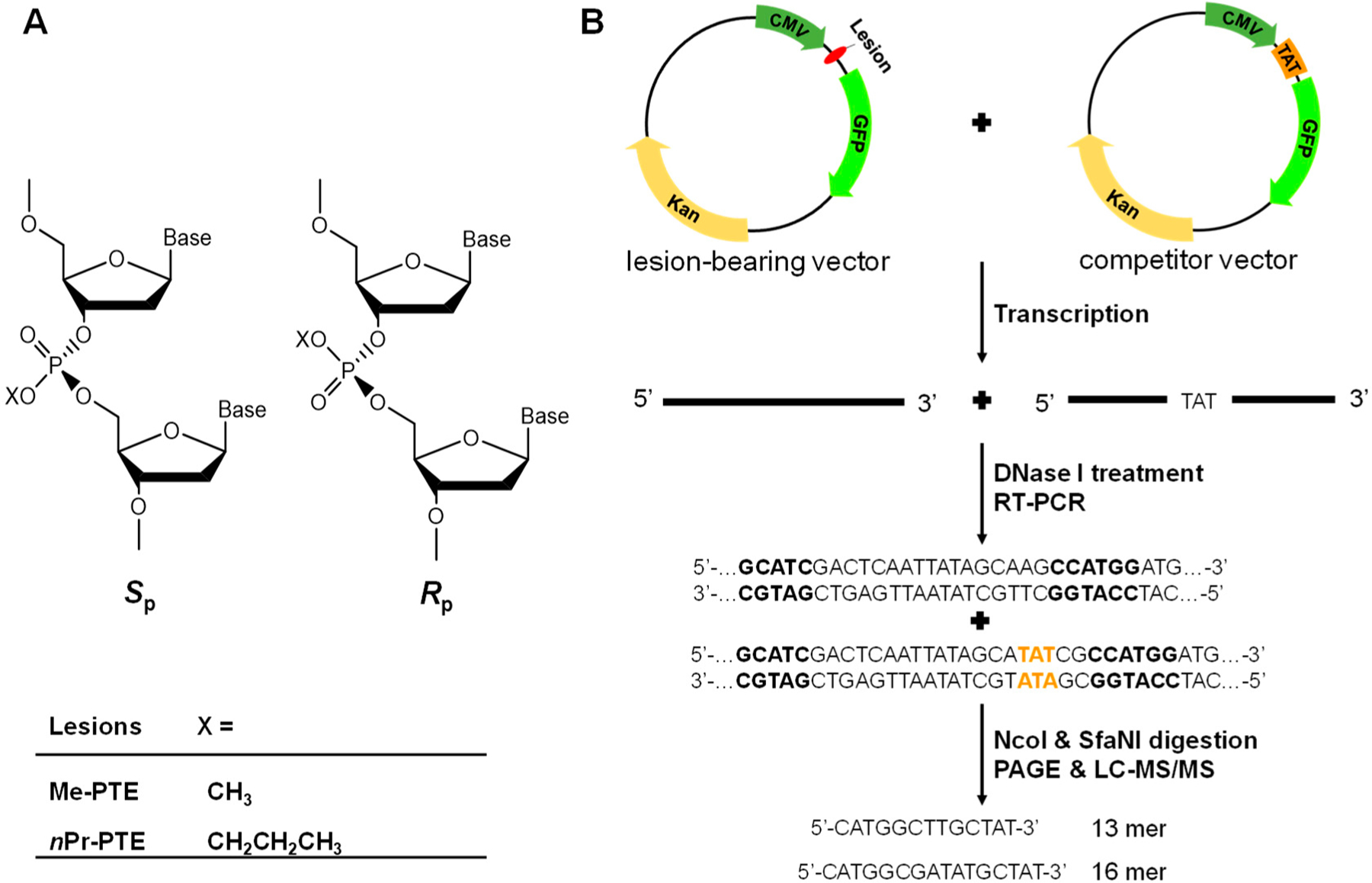
Schematic diagrams depicting the *S*_P_ and *R*_P_ diastereomers of Me-phosphotriester (PTE) and *n*Pr-PTE lesions in DNA (**A**) and the CTAB assay system (**B**).

Apart from nucleobases, alkylating agents can attack internucleotide phosphate group in DNA to yield alkyl phosphotriester (alkyl-PTE) lesions [11]. For example, 12–17% and 55–57% of the total alkylation products in duplex DNA treated with *N*-methyl-*N-*nitrosourea and *N*-ethyl-*N*-nitrosourea are Me- and Et-PTE, respectively [12]. Additionally, PHB-PTE adducts constitute 38–55% and 34–40% of all the measured pyridine-containing DNA lesions in lung and liver tissues, respectively, of rats exposed with nicotine-derived nitrosamine ketone (NNK) in drinking water [13]. Moreover, depending on which of the two non-carbon-bound oxygen atoms on the backbone phosphate is alkylated, alkyl-PTEs can form in the *S*_P_ or *R*_P_ configuration (Figure 1A) [11].

Alkyl-PTE lesions may be more difficult to repair than nucleobase alkylation products. For instance, in mouse liver tissues, Et-PTE lesions exhibited a longer half-life than any nucleobase ethylation products [14]. The poor repair of alkyl-PTE lesions suggests that they are likely encountered by DNA replication and transcription machineries [15]. Little, however, is known about how alkyl-PTE lesions compromise the efficiency and fidelity of DNA replication or transcription in human cells [11]. TLS is an important component of cells’ DNA damage tolerance system [16]. We observed recently that TLS polymerase (Pol) η could promote the transcriptional bypass of the minor-groove *N*^2^-alkyl-2′-deoxyguanosine adducts in human cells [17]. It remains unclear whether Pol η would also modulate the transcriptional bypass of alkyl-PTE lesions.

In the present study, we sought to fill in these knowledge gaps by investigating the transcriptional outcomes of alkyl-PTEs in mammalian cells, and the alterations of such outcomes by loss of function of some important TLS polymerases.

## 2. Experimental Procedures

Except otherwise specified, all chemicals were acquired from Sigma-Aldrich (St. Louis, MO, USA) or Thermo Fisher Scientific (Waltham, MA, USA), and all enzymes were from New England Biolabs (Ipswich, MA, USA). Turbo DNase and M-MLV reverse transcriptase were from Invitrogen (Waltham, MA, USA) and Promega (Madison, WI, USA), respectively. Lesion-free oligodeoxynucleotides (ODNs) used in this study were obtained from Integrated DNA Technologies (Coralville, IA, USA). [γ-^32^P] ATP was purchased from Perkin Elmer Life Sciences (Waltham, MA, USA).

The 12-mer ODNs carrying a site-specifically inserted Tp(alkyl)T (alkyl = Me or *n*Pr) were freshly synthesized following the same method as reported previously [18]. The HEK293T human embryonic kidney epithelial cells were obtained from ATCC and cultured in Dulbecco’s Modified Eagle’s medium supplemented with 10% fetal bovine serum. HEK293T cells depleted of Pol η, Pol ι, Pol κ, Pol ζ and REV1 were previously generated using CRISPR/Cas9 genome editing [19,20].

### 2.1. Construction of Transcription Templates

We adhered to previously described procedures [21,22] to prepare the non-replicative double-stranded vectors with an alkyl-PTE being inserted at a specific position. In brief, lesion-free control vector was modified from the non-replicating pTGFP-T7-Hha10 plasmid by EcoRI-NheI digestion, purification and ligation with a 50-mer ODN (5′-CTAGCGGATGCATCGACTCAATTATAGCAAGCCATGGATGACTCGCTGCG-3′) and its complementary strand. The control vectors were then nicked with Nt. BstNBI and annealed with a 25 mer complementary ODN (5′-CATCGACTCAATTATAGCAAGCCAT-3′) in excess to remove the cleaved 25 mer single-stranded ODN. Following purification of the gapped plasmids from the mixture by using a 100 kDa cutoff centrifugal filter, the 5′-phosphorylated 12 mer alkyl-PTE-containing ODN, 5′-ATGGCTp(x)TGCTAT-3′ (x = Me or *n*Pr), and 13 mer lesion-free ODN (5′-AATTGAGTCGATG-3′) were annealed into the gap. T4 DNA ligase was then added to seal the gap. Finally, the fully ligated, supercoiled lesion-containing plasmids were isolated from the ligation mixture by agarose gel electrophoresis and gel extraction.

### 2.2. Cellular Transcription, RNA Extraction and Amplification

Cellular transcription experiments were carried out as previously reported [21,22]. The lesion-containing plasmids and the lesion-free control plasmid were individually premixed with the competitor plasmid at a molar ratio of 5:1 (lesion/competitor) and 1:1 (control/competitor), respectively, and used as templates for transfection. HEK293T cells and the isogenic TLS polymerase-deficient cells were seeded (1 × 10^5^) in 24-well plates and cultured at 37 °C in 5% CO_2_ overnight, followed by transfection with 50 ng of the templates and 450 ng carrier plasmid (self-ligated pGEM-T, Promega) using TransIT-2020 (Mirus Bio, Madison, WI, USA) in accordance with the manufacturer’s instructions. After transfection, the transcripts of the mixed genomes were recovered from cells 24 h later using Total RNA Kit I (Omega Bio-tek, Norcross, GA, USA), and any remaining DNA was eliminated using a DNA-free kit (Ambion, Austin, TX, USA). The relevant transcripts were reverse transcribed and PCR-amplified, as previously described [21].

### 2.3. Restriction Digestion and Polyacrylamide Gel Electrophoresis (PAGE) Analysis

Prior to PAGE analysis, samples were prepared using restriction digestion with NcoI/SfaNI and post-labeling. In 10 μL of NEB buffer 3.1, 150 ng of the RT-PCR products from each sample were treated with 5 U NcoI and 1 U shrimp alkaline phosphatase (rSAP) at 37 °C for 1 h before being heated at 80 °C for 20 min. The newly released 5′-termini were then radiolabeled by adding 5 U T4 polynucleotide kinase and 1.66 pmol [γ-^32^P]ATP. The resulting mixture was heated at 75 °C for 20 min and subsequently digested with 2 U SfaNI in 20 μL of NEB buffer 3.1 at 37 °C for 1.5 h. The DNA mixture was resolved using 30% native PAGE (acrylamide/bis-acrylamide = 19:1) and quantified by phosphorimager after the reaction was terminated with 20 μL formamide gel-loading buffer (2×) [21]. The relative bypass efficiency (RBE) was calculated from the intensities of the radiolabeled DNA bands using the formula of RBE (%) = (lesion signal/competitor signal)/(control signal/competitor signal) × 100%, where the competitor signal served as the internal standard.

### 2.4. LC-MS/MS Analysis

The transcription products resulting from the alkyl-PTE-containing templates were identified using LC-MS and MS/MS, as previously described [21,22]. RT-PCR products were treated with 50 U NcoI and 20 U rSAP in 250 μL of NEB buffer 3.1 at 37 °C for 2 h, followed by heating at 80 °C for 20 min. SfaNI (50 U) was added to the resultant solution, and allowed to react with amplification products at 37 °C for 2 h before being extracted with phenol, chloroform, and isoamyl alcohol (25:24:1, *v*/*v*). To precipitate the DNA, 2.5 volumes of ethanol and 0.1 volume of 3.0 M sodium acetate were added to the collected aqueous phase. The DNA pellet was redissolved in water and used for LC-MS and MS/MS analysis. An LTQ linear ion trap mass spectrometer (Thermo Fisher Scientific) was set up in the negative-ion mode for monitoring the fragmentations of the [M-3H]^3−^ ions of the 13-mer ODNs, 5′-pCATGGCMNGCTAT-3′, where “MN” designates TA, TT, TC, or TG.

## 3. Results

Most human cells are not continuously proliferating or replicating but rather quiescent or slowly replicating in nature; therefore, maintaining faithful and efficient transcription is pivotal for normal cell function [23–25]. Recently, our laboratory developed a competitive transcription and adduct bypass (CTAB) assay for investigating transcriptional perturbations conferred by DNA lesions in vitro and in cells [20]. By using this assay (Figure 1), we investigated the impact of two pairs of alkyl-PTEs, i.e., the *S*_P_ and *R*_P_ diastereomers of T(Me)T and T(*n*Pr)T (Figure 1), on the efficiency and fidelity of transcription in mammalian cells. In this method, we prepared non-replicative double-stranded vectors harboring a single site-specifically incorporated alkyl-PTE as well as the corresponding non-lesion control and competitor vectors. Relative to the lesion-harboring or control plasmid, the competitor plasmid contains three additional nucleotides (Figure 1B) and serves as an internal reference for quantifying the extent to which a lesion in a double-stranded plasmid impedes cellular transcription. The alkyl-PTE lesions were placed downstream of the transcription start site of the cytomegalovirus (CMV) promoter. This design enables the examination about the effects of DNA lesions on transcription elongation mediated by mammalian RNA polymerase II (RNAPII).

**Figure 2.**
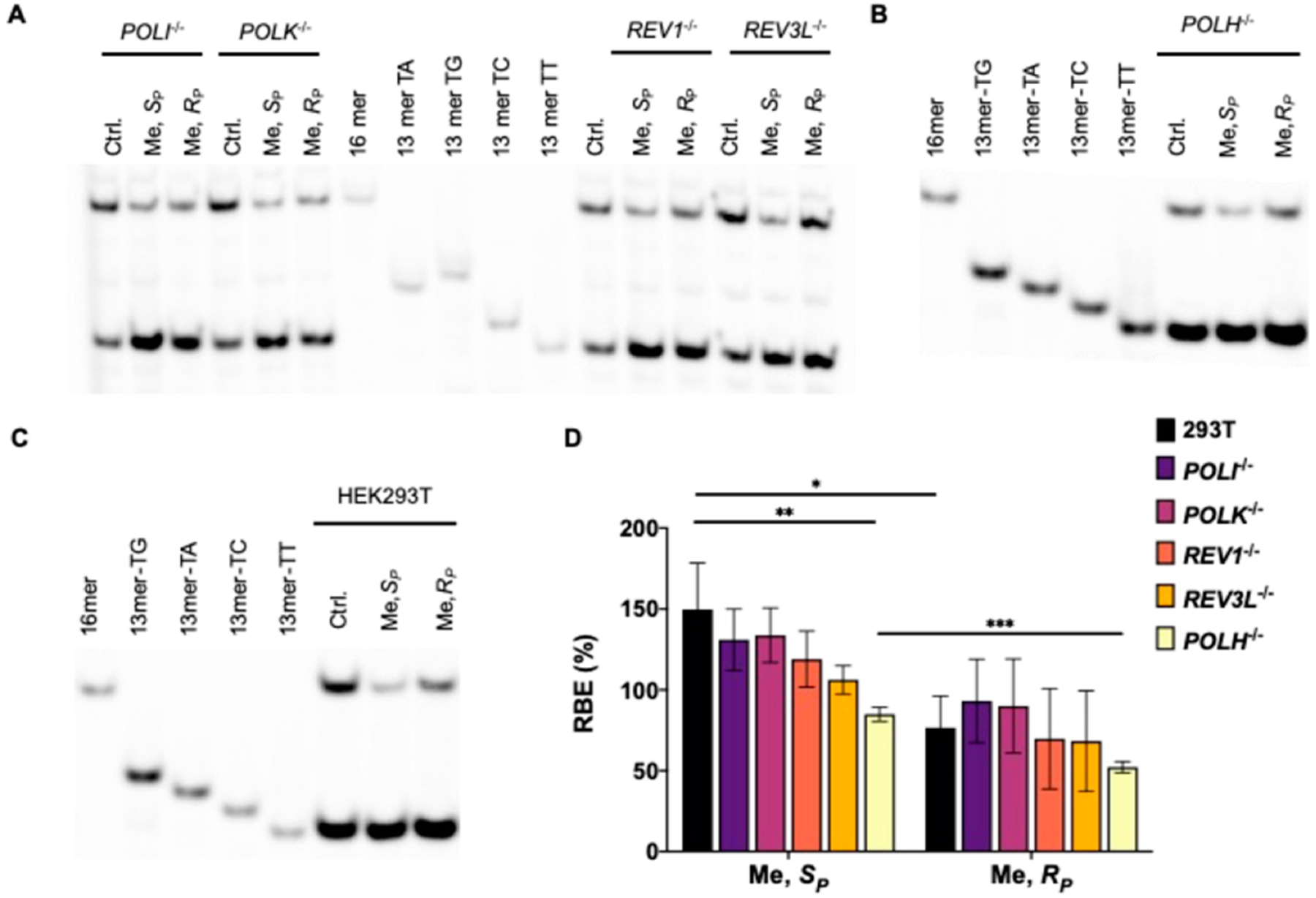
Transcriptional bypass of Me-PTE in HEK293T cells and isogenic cells depleted of TLS polymerases. (**A**–**C**) Gel image showing the 16 mer (from the competitor genome) and 13 mer (from the control or lesion-containing genome) digestion products formed from the original template strand, where 13 mer TG/TA/TC/TT designate [5′-^32^P]-labeled standard ODNs 5′-CATGGCMNGCTAT-3′, with ‘MN’ being TT, TG, TC and TA, respectively. (**D**) The relative bypass efficiencies (RBE) of the two diastereomers of Me-PTE lesions in HEK293T cells and the isogenic cells depleted of TLS polymerases. The data represent mean ± S.D. (n = 3). *, 0.01 < *p* < 0.05; **, 0.001 < *p* < 0.01; ***, 0.0001 < *p* < 0.001. The *p* values were calculated by using Sidek’s multiple comparisons test.

In the CTAB assay (Figure 1B), the lesion-containing or undamaged control plasmids are pre-mixed individually with the competitor vector at specific molar ratios and used as templates for cellular transcription. The resulting transcripts are isolated and incubated with RNase-free DNase I to eliminate residual contaminating DNA template. The ensuing runoff transcripts are reverse transcribed to produce cDNA, which is then PCR amplified. The PCR amplicons are subsequently digested with two restriction enzymes, and the digestion products are subjected to PAGE and LC-MS/MS analyses [21]. The degree through which a DNA adduct impedes transcription, represented as RBE, is determined from the initial genome ratios employed for the transfection and the ratio of the amounts of transcription products formed from the damage-containing genome (i.e., the 13mer products shown in Figure 2) over that from the competitor genome (i.e., the 16mer product, Figure 2), where we also take into account the corresponding ratio obtained for the control damage-free genome over the competitor genome.

### 3.1. Effects of Alkyl-PTE Lesions on Transcription in Mammalian Cells

**Figure 3.**
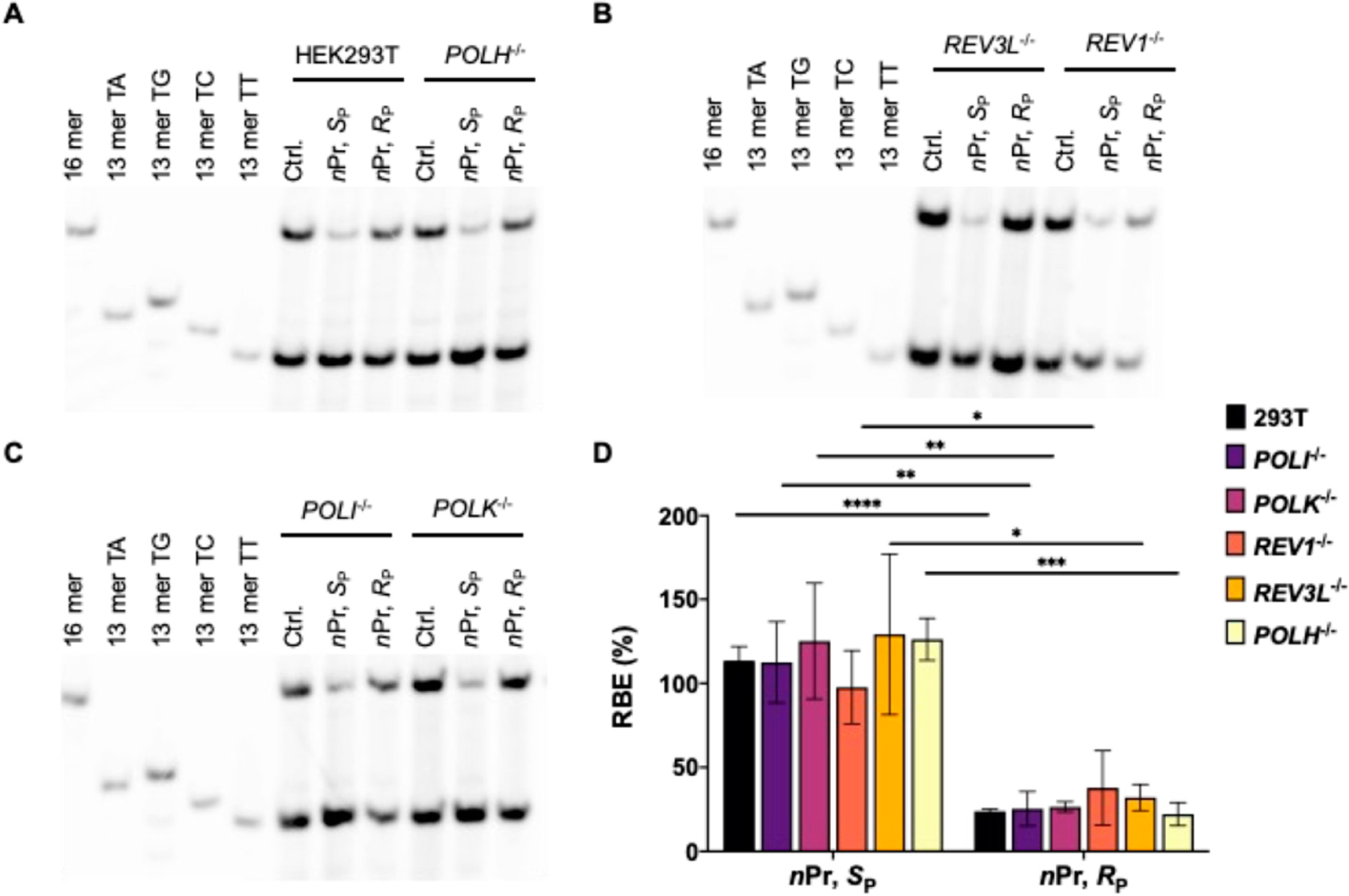
Transcriptional bypass of *n*Pr-PTE in HEK293T cells and isogenic cells depleted of TLS polymerases. (**A**–**C**) Gel image showing the 16 mer (from the competitor genome) and 13 mer (from the control or lesion-containing genome) digestion products formed from the original template strand, where 13 mer TG/TA/TC/TT designate [5′-^32^P]-labeled standard ODNs 5′-CATGGCMNGCTAT-3′, with ‘MN’ being TT, TG, TC and TA, respectively. (**D**) The RBE values of the two diastereomers of *n*Pr-PTE lesions in HEK293T cells and isogenic cells depleted of TLS polymerases. The data represent the means ± S.D. (n = 3). *, 0.01 < *p* < 0.05; **, 0.001 < *p* < 0.01; ***, 0.0001 < *p* < 0.001; ****, *p* < 0.0001. The *p* values were calculated by using Sidek’s multiple comparisons test.

**Figure 4.**
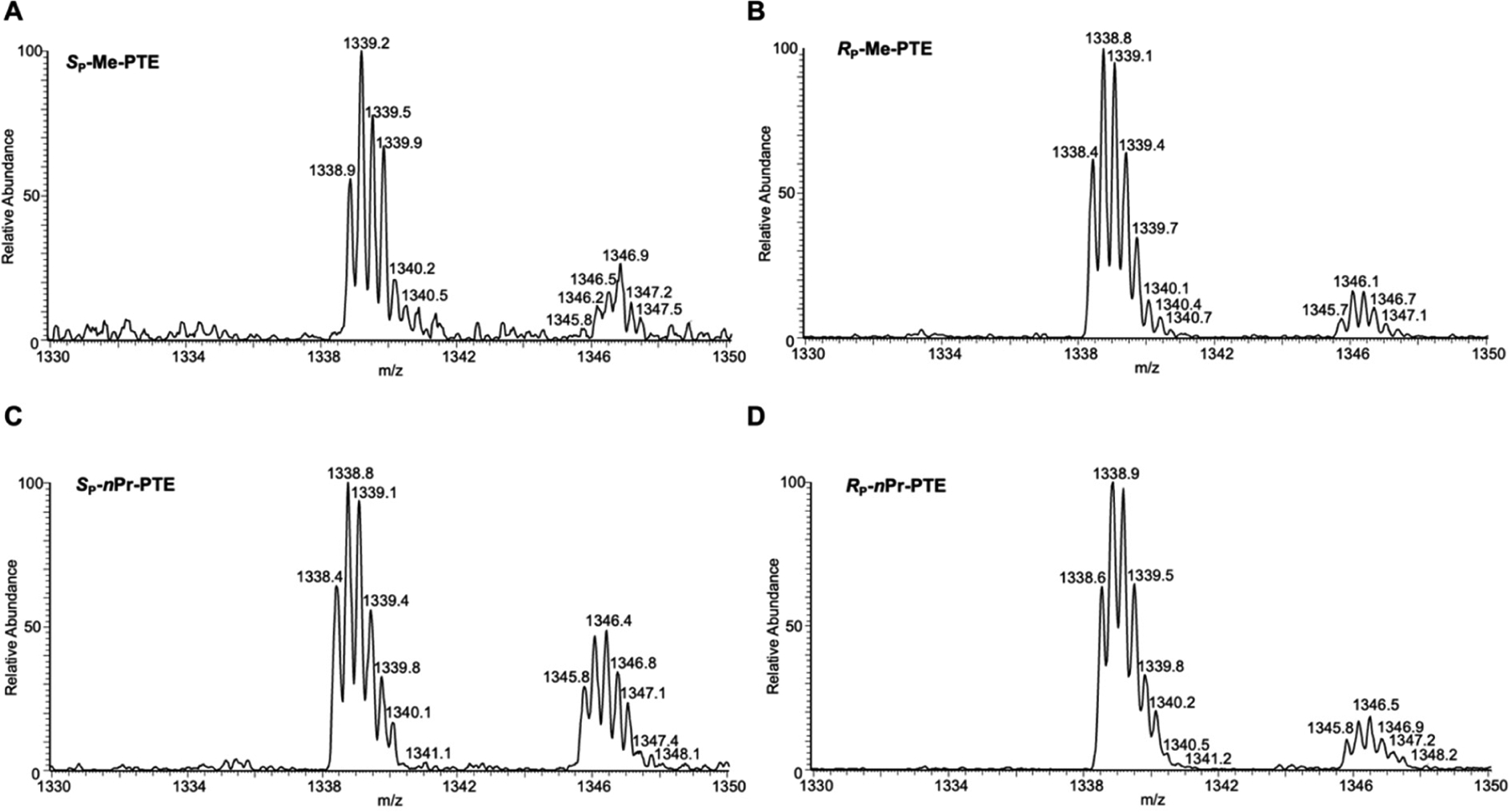
Higher-resolution “zoom-scan” ESI-MS for monitoring the [M-3H]^3−^ ions of the restriction fragments of interest, i.e., 5′-pCATGGCMNGCTAT-3′ (MN = TT, TG, TC and TA) from the transcription of *S*_p_-Me-PTE (**A**), *R*_p_-Me-PTE (**B**), *S*_p_-*n*Pr-PTE (**C**) and *R*_p_-*n*Pr-PTE (**D**) in HEK293T cells. The isotope cluster at *m/z* 1339 corresponds to the [M-3H]^3−^ ions of the non-mutagenic product, 5′-pCATGGCTTGCTAT-3′. The isotope cluster at *m/z* 1346 corresponds to the [M-4H+Na]^3−^ ions of the non-mutagenic product. The calculated *m/z* values for the putative mutagenic products of TG, TC and TA are 1347, 1333.5 and 1341.5, respectively.

**Figure 5.**
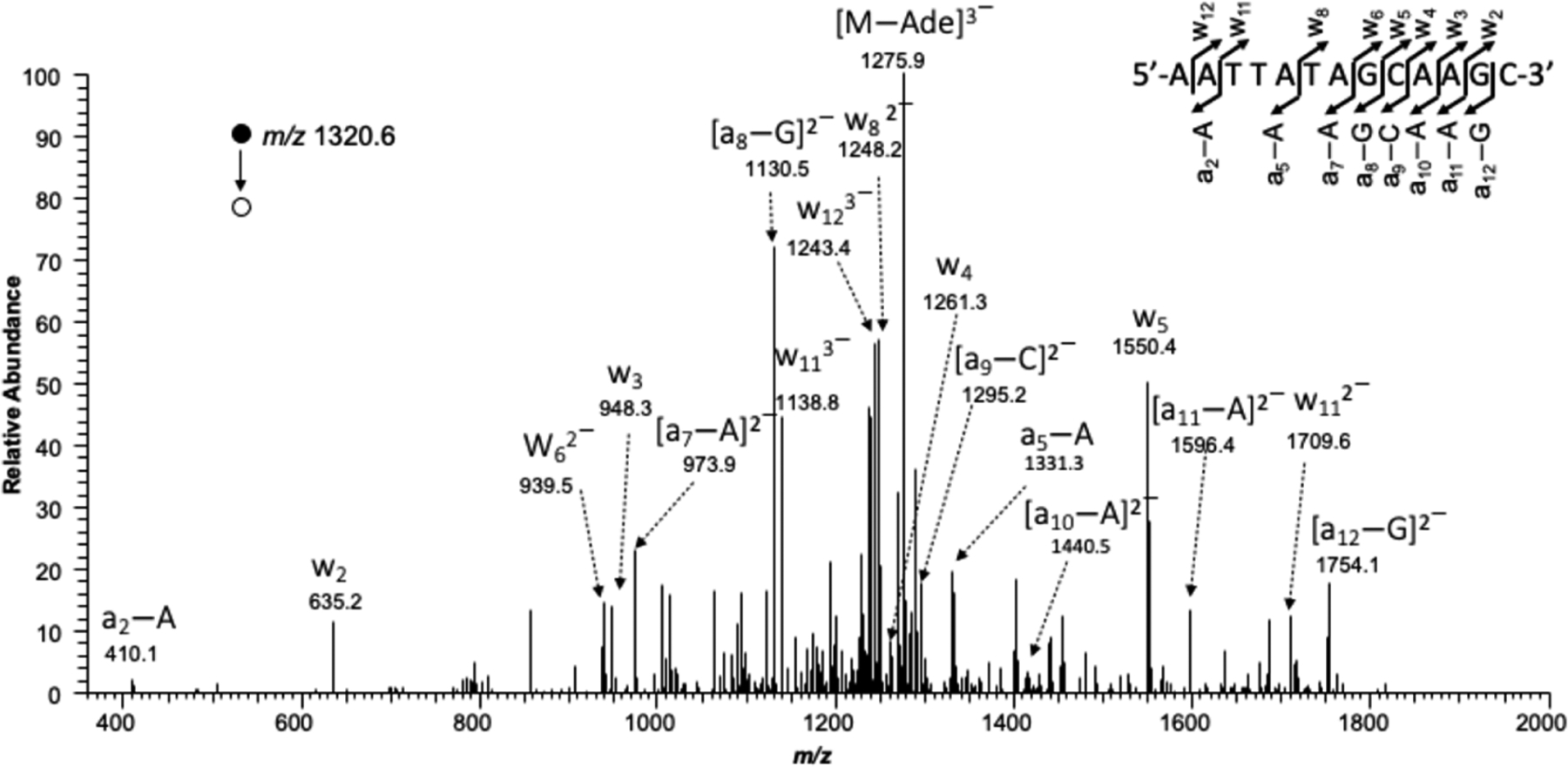
MS/MS for confirming the restriction fragment corresponding to wild-type transcript arising from alkyl-PTE containing substrates.

LC-MS/MS and PAGE analyses for the restriction fragments of RT-PCR products showed that the addition of alkyl groups to the phosphate backbone did not perturb transcription fidelity, as manifested by the absence of mutagenic transcription products for the PTE adducts examined (Figures 2–5). The efficiencies of transcription, however, were differentially affected by the stereochemical configurations of the alkyl groups. In HEK293T cells, while the *S*_P_ diastereomers of the Me- and *n*Pr-PTE lesions are well tolerated, the *R*_P_ diastereomer of the two lesions constitute moderate and strong impediments to transcription elongation, respectively (Figures 2 and 3).

### 3.2. Pol η Promotes the Transcription of S_P_-Me-Alkyl-PTE Lesions in Mammalian Cells

DNA polymerase (Pol) η is a Y-family TLS polymerase recently shown to support the transcriptional bypass of *N*^2^-alkyl-2′-deoxyguanosine (*N*^2^-alkyl-dG) lesions [17]. To examine whether Pol η or other TLS polymerases can modulate the transcription across the alkyl-PTE lesions, we performed the aforementioned CTAB assay in TLS polymerase-knockout cell lines, which were previously generated through CRISPR/Cas9 and single colony isolation from HEK293T cells [19,20]. We observed that genetic ablation of *POLH* in HEK293T cells conferred a substantial diminution in RBE for *S*_P_-Me-PTE, whereas the corresponding change in RBE for *R*_P_-Me-PTE was very subtle (Figure 2). However, loss of Pol η did not influence the transcriptional efficiency of *n*Pr-PTEs lesions (Figure 3). Likewise, genetic depletion of Pol ι, Pol κ, Pol ζ or REV1 did not affect appreciably the efficiencies in transcriptional bypass of any of the alkyl-PTE lesions (Figures 2 and 3).

## 4. Discussion

We systematically investigated, for the first time, how alkyl-PTE lesions compromise the efficiencies and fidelities of transcription in mammalian cells. We found that transcription fidelity is not perturbed by alkyl-PTEs as transcription over alkyl-PTEs is error-free. Previously, our laboratory showed that replication across *R*_P_-Me-PTE and both diastereomers of *n*Bu-PTEs is error-free in *Escherichia coli* cells, whereas replicative bypass of the *S*_P_-Me-PTE formed at XT sequence context (X = A, T, C, G), which is a cognitive substrate for *Ada* protein, is error-prone [26,27]. There is, however, no mammalian ortholog of *Ada* with a methylphosphotriester-DNA methyltransferase activity [28]. We observed that the transcript yields for the alkyl-PTE lesions are affected by the size and stereochemistry of the alkyl groups. The *R*_P_ diastereomers Me- and *n*Pr-PTE impose moderate and strong blockages on transcription, respectively; the *S*_P_-*n*Pr diastereomers of the two lesions are, however, well tolerated (Figures 2 and 3). It was reported that many of the small non-bulky DNA adducts, including thymidine glycol, abasic site, *O*^6^-methylguanine and 8-oxo-7,8-dihydro-2′-deoxyguanosine (8-*oxo*-dG), did not block RNA polymerase. [29–34] On the contrary, the strong helix-distorting bulky adducts can completely hinder RNAP II from forward translocation [35,36].

Our observation of the pivotal effects of stereochemical configuration of alkyl-PTE lesions on transcriptional bypass efficiencies in human cells is reminiscent of the influence of stereochemical configuration of the alkyl-PTEs on DNA replication in *E. coli* cells, where the *R*_P_ diastereomers were replicated at markedly lower efficiencies than the *S*_P_ counterparts [18,26,27]. These results suggest that the alkyl-PTE lesions in the *S*_P_ configuration, with the alkyl group pointing perpendicular out from the phosphate backbone [11], are in favor of interacting with DNA and RNA polymerases, whereas the lesions in the *R*_P_ configuration, with the alkyl group projecting into the major groove of DNA double helix [11], hinder damage bypass mediated by both DNA and RNA polymerases. It will be important to assess, by employing molecular modeling and structural biology tools, the molecular basis underlying the effects of the size and stereochemistry of the alkyl-PTE lesions on transcriptional bypass in the future.

Alkyl-PTEs are known to be poorly repaired in mammalian tissues; hence, these lesions may be encountered by DNA replication and transcription machineries. Our result suggested that Pol η may be able to assist transcriptional bypass of the *S*_P_-Me-PTE lesion. This result parallels our recent finding that Pol η promotes the transcriptional bypass of *N*^2^-alkyl-dG lesions in human cells [17]. Among all the TLS polymerases, Pol η is especially versatile. Deficiency in this polymerase is known to contribute to the variant form of xeroderma pigmentosum (XPV) [37], and this is due to Pol η’s role in efficient and accurate bypass of the UV-induced cyclobutane pyrimidine dimer (CPD) during replication [38]. In addition, Pol η is able to robustly insert and elongate with the correct rNTP opposite CPD-containing template, [39,40] and the polymerase is responsible for inserting consecutive ribonucleotides into genomic DNA [40–42]. Moreover, *Saccharomyces cerevisiae* Pol η extends the RNA primer with rNTPs an order of magnitude more efficiently than inserting rNTPs into DNA [43]. Moreover, Pol η’s large active site can accommodate RNA/DNA, DNA/DNA or DNA/RNA duplex [40]. It is therefore tempting to speculate that Pol η may assist RNAPII in transcription across small unrepaired PTE lesion. Although our results cannot directly exclude the possibility that Pol η may participate in repair, it was previously demonstrated that Pol η’s recruitment to UV-induced damage sites outside of S phase was independent of nucleotide excision repair in human cells [44] Another specialized DNA polymerase, Pol β, can incorporate rCTP opposite 8-*oxo*-dG [45] and it will be interesting to explore whether other polymerases, including Pol β, also contribute to transcriptional bypass of alkyl-PTE lesions.
